# Ethyl Acetate Extract of *Selaginella doederleinii* Hieron Induces Cell Autophagic Death and Apoptosis in Colorectal Cancer *via* PI3K-Akt-mTOR and AMPKα-Signaling Pathways

**DOI:** 10.3389/fphar.2020.565090

**Published:** 2020-10-19

**Authors:** Shaoguang Li, Xuewen Wang, Gang Wang, Peiying Shi, Shilan Lin, Dafen Xu, Bing Chen, Ailin Liu, Liying Huang, Xinhua Lin, Hong Yao

**Affiliations:** ^1^Department of Pharmaceutical Analysis, School of Pharmacy, Fujian Medical University, Fuzhou, China; ^2^Higher Educational Key Laboratory for Nano Biomedical Technology of Fujian Province, Fujian Medical University, Fuzhou, China; ^3^Nano Medical Technology Research Institute, Fujian Medical University, Fuzhou, China; ^4^Department of Traditional Chinese Medicine Resource and Bee Products, Bee Science College, Fujian Agriculture and Forestry University, Fuzhou, China; ^5^Fujian Key Laboratory of Drug Target Discovery and Structural and Functional Research, Fujian Medical University, Fuzhou, China

**Keywords:** colorectal carcinoma, autophagy, apoptosis, *Selaginella doederleinii* Hieron ethyl acetate, antitumor mechanism

## Abstract

Colorectal cancer is one type of cancer with high incidence rate and high mortality worldwide. Thus, developing new chemotherapeutic drugs is important. The *Selaginella doederleinii* Hieron ethyl acetate (SDEA) extract showed good anti-colon cancer effect *in vitro* and *in vivo*, but its mechanism is unclear. This study aimed to further reveal the anti-colon cancer effect of SDEA and its possible mechanism. The effects on cell viability, apoptosis, autophagy, and cell cycle in colorectal cells (HT29 and HCT116) were studied using MTT assay, fluorescence microscopy, transmission electron microscopy, and flow cytometry. The mechanisms were further studied using cell transfection, Western blot, and real-time quantitative polymerase chain reaction assay. The effect of xenotransplantation *in vivo* was observed using immunohistochemistry. Results showed that SDEA inhibited cell proliferation and induced cell morphological changes, cell cycle arrest, autophagy, and apoptosis. It also induced loss of mitochondrial membrane potential, increased the autophagic flux, raised the ratio of Bax/Bcl-2, activated caspases, and inhibited PI3K-Akt-mTOR signaling pathways. Furthermore, SDEA inhibited the growth of xenograft tumors in a dose-dependent manner. Immunohistochemistry analysis confirmed the alteration of autophagy- and apoptosis-related proteins and immunohistochemical microvascular density in xenografts, which were consistent with the results *in vitro*. Therefore, SDEA is important for developing candidate drugs against colorectal cancers.

## Introduction

Colorectal cancer has an estimated incidence of over one million new cases annually worldwide. According to the Global Cancer Statistics 2018, colorectal cancer is the fourth leading cause of death, accounting for 5.8% of all sites (36 cancers). Colon cancer more often affects people in well-developed countries than those in less developed countries (Bray et al., 2018). Screening and developing novel anti-colon cancer chemotherapeutic agents remain as hot issues. Many kinds of natural-derived anticancer agents (e.g., paclitaxel, camptothecin, and their derivatives) have been developed and widely used in recent decades to treat several types of cancer in clinical practice (Moosavi et al., 2018). Screening anti-cancer candidates from natural products, especially those based on folk traditional experiences on natural anticancer remedies, is considered an efficient method to develop novel chemotherapeutic agents.

*Selaginella doederleinii* (*SD*) Hieron, a species that belongs to the *Selaginella* family, is an important object of research. In clinical application, *SD* is commonly used in several famous antitumor prescriptions (such as TCM Yiqi Yangyin for treatment of lung cancer), or has been prepared into tablets (mainly consisting of *SD* alcohol extract) for the treatment of digestive tract cancer, nasopharynx cancer, and lung cancer. Modern pharmacological investigations confirmed that the antitumor activity of *SD* extract could effectively inhibit the proliferation of human breast cancer MCF-7 cells, human lung cancer A549 cells (Sui et al., 2016), and human nasopharyngeal carcinoma CNE1 and CNE2 cells *in vitro* (Liu et al., 2011; Lian et al., 2013). *SD* also inhibits various tumor cell-related enzymes, such as protein kinase C and DNA polymerase, tumor growth (such as Lewis and NPC TW03 cells) (Yao et al., 2017), and metastasis (such as B16F-10 cells) (Guruvayoorappan and Kuttan, 2007) *via* HPLC *in vivo*. In previous studies, 20 flavonoid components were enriched in the extract of *SD* by ethyl acetate (SDEA) (Li et al., 2014; Yao et al., 2017). Thus, SDEA extract was prepared in the present study. This extract could inhibit the growth of different kinds of cancer cells, such as lung cancer cells (A549, PC-9, and NCI-H460) (Banerjee et al., 2002; Cao et al., 2010; Tsui et al., 2014; Jung et al., 2017; Sui et al., 2017), nasopharyngeal carcinoma cells (CNE2), hematological neoplasms cells (HL60 and K562) (Li et al., 2014), human breast cancer cells (MCF-7) (Pei et al., 2012; Chen et al., 2015), hepatoma cells (HpG2 and SMMC-7721) (Zheng et al., 2016; Liu et al., 2019), and colon cancer cells (HT29, SW620, and SW480) (Kuete et al., 2016; Lee et al., 2018; Zhang et al., 2014). In particular, SDEA has a significant inhibitory effect on human colorectal cancer cells HT29 and HCT1116. However, no report is available on the anti-colon cancer effect and mechanism of SDEA, thereby hindering further development of the SDEA extract for medicinal usage.

This paper aimed to explore the role and mechanism of SDEA in colon cancer. On the basis of previous studies, five human colon cancer cells were used to further evaluate the in-vitro anti-colon cancer activity of the SDEA extract. The cell lines most sensitive to SDEA were subsequently selected to study the effect of the SDEA extract on apoptosis and autophagy and reveal its possible mechanism against colon cancer. Meanwhile, a colon cancer cell xenograft tumor model was used to study the effect of SDEA against colon cancer *in vivo*.

## Materials and Methods

### Plant Materials

All *SD* specimens bought from Xiyang drugstore were identified and authenticated by Professor Hong Yao. Voucher specimens (no. 1608FZ) were deposited in Room 312 of Department of Pharmaceutical Analysis, and the herbarium code of the herbarium is SD1608FZ. Plant herbs were chopped and extracted with 70% ethanol (Sinopharm Chemical Reagent Co., Ltd, no. 20170718). The ethanol extract was concentrated *via* rotary evaporation under reduced pressure to remove the ethanol. The concentrate was then suspended with water and successively extracted with petroleum ether, dichloromethane, and ethyl acetate. The ethyl acetate extracts were concentrated and stored at 4°C for the next test.

HPLC analysis was performed on Shimadzu HPLC 20A system (UV detector, 288 nm) and a SinoChrom RD C18 column (150 × 4.6 mm, 5 μm, Sino-chromatogram Sci & Tech, Inc.). The mobile phase was comprised of (A) aqueous acetic acid (0.5%, v/v) and (B) acetonitrile using a gradient elution of 10–45% in 0–25min, 45–58% in 25–45 min, 58–95% in 45–46 min, and 100% in 46–51 min. The re-equilibration time was 10 min, giving a total run time of 51 min. The flow rate was 1.0 mL/min and the injection volume was 10 μL.

### Cell Lines and Reagents

Five colon cancer cells, including HT29, HCT116, SW620, SW480, and SW1116, were used for cytotoxicity evaluation to investigate the in-vitro activity of SDEA on colorectal cancer. Colorectal cancer cell lines were purchased the from Chinese Academy of Sciences Shanghai Cell Bank (Shanghai, China) in October 2016, cultured in high-glucose (25 mM) DMEM medium containing 10% fetal bovine serum (Gibco, no. 1830857) and 100 U/mL penicillin/streptomycin, and incubated in 5% CO_2_ and 37°C incubator. Cell viabilities were determined using MTT assay (Aladdin, Aladdin reagent (Shanghai) Co., Ltd, no. 20170728) in accordance with the manufacturer’s instructions.

In a 96-well plate, 200 μL of cell suspension (4 × 10^3^ cells) was added into each well and incubated in a 37°C incubator with 5% CO_2_ for 24 h until cell adherence was complete. Fresh medium containing different SDEA concentrations was replaced for each well, and more than five parallel wells were set up for each concentration. After treatment with different concentrations (10–200 μg/mL or 12.5–200 μg/mL) and times (12, 24, and 48 h), MTT solution was added, and the absorbance in each well was measured at 570 nm after 4 h. The inhibitory rates were calculated by the absorbance values (OD values) of the treatment and the control groups (Kukulakoch et al., 2018).

$$\mathrm{Inhibitory rate}\;(\%)=(OD_{\text{control}}-OD_{\text{sample}})/(OD_{\text{control}}-OD_{\text{blank}})\times 100.$$

### Ultramorphological Analysis by Transmission Electron Microscopy

Cells treated with SDEA were fixed in modified Karnovsky’s fixative (1.5% glutaraldehyde, 3% paraformaldehyde, and 5% sucrose in 0.1 M cacodylate buffer at pH 7.4) for 12 h at 4°C, followed by treatment with 1% osmium tetroxide in 0.1 M cacodylate buffer for an additional 2 h at 4°C. The cells were then stained in 2% uranyl acetate for 20 min and dehydrated in ethanol and acetone. The samples were embedded in epoxy resin, sectioned (60–70 nm), and placed on formvar and carbon-coated copper grids. These grids were stained with uranyl acetate and lead citrate, and images were obtained using a transmission electron microscope (FEI, TECNAI G^2^, USA) (Jin et al., 2020).

### Mitochondrial Membrane Potential (MMP, Δφ_m_) Analysis

Abnormal MMP activates the mitochondrial apoptotic pathway and induces apoptosis. JC-1 is sensitive to MMP. In this study, it was used to measure the MMP of colon cell lines after being treated with SDEA. The methods were according to the guideline of JC-1 probe kits purchased from KeyGen Biotech. Co., Ltd (Nanjing, China).

The cells were collected, fully blown into single cell suspension, counted, and evenly inoculated into 12-well plates in accordance with the number of cells per well. When cell suspension was added, a small amount of culture medium was dripped at the place where the climbing piece was prepared to be placed in each hole. Then, the climbing piece was placed on the droplet and pressed for the climbing piece and the culture dish to be bonded together by the tension of the culture medium. Cell suspension was added to prevent the climbing piece from floating, resulting in double-layer cell patch. Different SDEA concentrations (0, 20, 40, 60, 80, and 100 μg/mL) were then added and treated for 6, 12, and 24 h. Afterward, the washed cells were trypsinized and resuspended in 0.5 mL of incubation buffer, and 0.5 mL of JC-1 staining working fluid (100× or 200×) was added. After 15–20 min of incubation at 37°C in the dark, the supernatant was immediately removed *via* centrifugation. Then, the stained cells were resuspended and immediately observed on a ﬂuorescence inverted microscope (DMI3000 B, Leica, Germany). The percentage of green ﬂuorescence from JC-1 monomers was used to demonstrate the cells that lost Δφ_m_ (Zhang et al., 2019).

### Apoptotic Detection

In this study, double fluorescent probes containing FITC Annexin V and PI were used in flow cytometry assays to reveal the cell inhibition mechanisms of HT29 and HCT116 cells with SDEA.

The cells in good or logarithmic growth state were evenly laid in six-well plates overnight. After cell adherence, the old culture medium was sucked up. After 0, 36, and 48 h, the cells were digested with 0.25% trypsin (without EDTA) and collected with a complete culture medium containing 0, 20, 40, 80, and 100 μg/mL of SDEA. Then, the cells were stained in accordance with the guideline of the FITC Annexin V apoptosis detection kit I (BD, no. 556547). After centrifugation, washing, debris removal, and dilution, the cells were added with 5 μL of Annexin V-FITC and 5 μL of PI, followed by gentle whirlpool, incubation at room temperature (25°C), and keeping in the dark for 15 min.

The cells without treatment were divided into three groups: the first group was added with PBS, the second group was added with 5 μL of Annexin V-FITC (no PI), and the third group was added with 5 μL of PI (no Annexin V-FITC). Incubation was conducted at room temperature (25°C) for 15 min in the absence of light. These three groups were used as control groups to regulate fluorescence compensation and set quadrants. After staining, analysis was performed *via* flow cytometry (BD, CSVerse) within 1 h (Ding et al., 2019).

### Flow Cytometric Analysis of Cell Cycle

The effects of different doses of SDEA on the cell cycle of HT29 and HCT116 cells were investigated. The cells were treated as follows: one group was intervened for 48 h with different SDEA concentrations (0, 20, 60, and 100 μg/mL), and the other group was treated with 100 μg/mL at different times (0, 12, 24, and 48 h). Then, the cells were digested, washed with 75% alcohol, and pre-cooled at 4°C. The cells were slowly added to the cell precipitation and fixed overnight at 4°C. The fixed cells were washed, and the residual fixative was removed. PI/RNase staining buffer (0.5 mL) was added, followed by incubation at room temperature and under light for 15 min. Afterward, the cells were stored in ice in the dark and detected using flow cytometry within 1 h (Jung et al., 2020).

#### Immunoblotting Assays

The autophagy- and apoptosis-related pathways were detected to reveal the mechanism of inhibition in colorectal carcinoma HT29 and HCT116 cells interfered by SDEA. After the cells were treated with the extract under different conditions, they were treated for protein extraction, which was performed using direct lysis with 1× RIPA cell lysis buffer (Beyotime Biotechnology, no. P0013K), and 1× protease and phosphatase inhibitor (Beyotime Biotechnology, cocktail for general use, MS-safe, 50×, no. P1048) were added immediately before use. The samples were boiled with added 5× SDS sample buffer for 10 min at 100°C and resolved using SDS–PAGE (Zhao et al., 2020).

The following antibodies were used: anti-ULK1 (1:1000, no. 8054S), Beclin 1 (1:1000, no. 3495S), SQSTM1/P62 (1:500, no. 8025S), LC3 A/B (1:1000, no. 4108S), p-Akt (1:1000, no. 4060S), cleaved-Caspase-9 (1:1000, no. 7237S), Caspase-3 (1:1000, no. 9665S), Bax (1:1000, no. 2774S), Bcl-2 (1:1000, no. 2870S), and Rabbit IgG (1:1000, no. 7074P2). All antibodies were purchased from Cell Signaling Technology.

#### Real-Time PCR Analysis

For qRT-PCR, the total RNAs from the harvested cells were prepared using TRIzol Reagent (Invitrogen, Carlsbad, CA, USA) in accordance with the manufacturer’s instructions. RNA concentrations were determined using a spectrophotometer (NanoDrop 2000, Thermo Fisher Scientific, USA). The quality of the RNA samples was ensured to have an A260/A230 ratio > 1.7 and A260/A280 ratio between 1.8 and 2.0. Approximately 1 μg of total RNA was subjected to RT-PCR using Revert Aid First Strand complementary DNA (cDNA) synthesis kit (Thermo Scientific, no. K1622). The messenger RNA (mRNA) expression levels of *Caspase-3*, *Bax*, *Bcl-2*, *LC3*, *P62*, *Beclin 1*, and *β-actin* were measured using RT-PCR. The primers used in the PCR were as follows:

*β-actin* forward: 5′- CACCCAGCACAATGAAGATCAAGAT -3′, reverse: 5′-CCAGTTTTTAAATCCTGAGTCAAGC -3′, *Caspase-3* forward: 5′-TGGAAGCGAATCAATGGACTCT -3′, reverse: 5′- TGAATGTTTCCCTGAGGTTTGC -3′, *Bax* forward: 5′- TTTTGCTTCAGGGTTTCATCCA -3′, reverse: 5′- TGCCACTCGGAAAAAGACCTC -3′, *Bcl-2* forward: 5′- ATCGCCCTGTGGATGACTGA -3′, reverse: 5′- GAGACAGCCAGGAGAAATCAAAC -3′*, LC3* forward: 5′- AGCAAAATCCCGGTGATCATC -3′, reverse: 5′- GCCGGATGATCTTGACCAACT -3′*, P62* forward: 5′- CAGTCCCTACAGATGCCAGAAT -3′, reverse: 5′- GCCGCTCCGATGTCATAGTT -3′*, Beclin 1* forward: 5′- GAGCCATTTATTGAAACTCCTCG -3′, and reverse: 5′ - CCCAGTGACCTTCAGTCTTCG -3′.

The annealing temperatures and the thermal cycles for *β-actin*, *Caspase-3*, *Bax*, *Bcl-2*, *LC3*, *P62*, and *Beclin 1* were 60°C and 40 cycles. For each cDNA, the mRNA level of the target gene was normalized to *β-actin* mRNA level. The results were expressed as the ratio of the normalized mRNA level of the target gene in cells treated with the SDEA to that in the control group. The experiments were performed in triplicate.

### Tandem mRFP-GFP Fluorescence Microscopy

A ﬂuorescence assay designed to monitor ﬂux relies on the use of a tandem monomeric RFP-GFP-tagged LC3. The GFP signal is sensitive to the acidic and/or proteolytic conditions of the lysosome lumen, whereas mRFP is more stable. Therefore, the colocalization of GFP and mRFP ﬂuorescence indicates a compartment, such as a phagophore or an autophagosome, that has not fused with a lysosome. By contrast, an mRFP signal without GFP corresponds to an amphisome or an autolysosome. The cells were infected with appropriate amounts of adenovirus carrying mRFP-GFP-LC3 to express the close-to-endogenous level of tandem mRFP-GFP-LC3 for 48 h, providing a convenient way to monitor the autophagic ﬂux in many cell types. After SDEA treatment, the cells were ﬁxed with 4% paraformaldehyde for 20 min and rinsed with PBS twice. They were mounted and visualized under a confocal microscope (Olympus FV-1,000). The total number of cells on the images was determined by nucleus staining with 4,6-diamidino-2-phenylindole. Autophagosome maturation was assessed by transfecting the mRFP-GFP-LC3 tandem vector. The percentage of red-only puncta was quantitated (Chen et al., 2019).

### In Vivo Xenograft Analysis

Previous studies have shown that SDEA has a significant proliferation inhibition activity on human colon cancer HT29 and HCT116 cells *in vitro*. In the present study, the anti-tumor activity *in vivo* was further evaluated by establishing subcutaneous transplantation models of colon cancer cells in nude mice (nu/nu, 6-week old males, License: scxk (Shanghai) 2017-0005, Slake Experimental Animal Co., Ltd, Shanghai), which were injected subcutaneously with 5 × 10^6^ HT29 or HCT116 stable cells in the right ﬂank region. When the tumor cells were transplanted subcutaneously into the nude mice to form tumors, the tumor volume was measured every 5 days using the formula (tumor volume = 1/2 [L × W^2^]). When the tumor volume reached approximately 100 mm^3^, the nude mice were randomly divided into five groups, including one negative control group, one positive control group, and three drug treatment groups. The positive control group was treated with 5-FU (i.v., 5 mg/kg/2 days). The drug treatment groups were treated with SDEA (po., 100, 200, and 300 mg/kg/day). The mice were killed on day 25, and the tumors were dissected and analyzed. The inhibition rate *in vivo* was counted as follows:

$$\text{Inhibitory rate}\;(\%)=({\text{Control}}_{\text{tumor weight}}-{\text{Intervention}}_{\text{tumor weight}})/{\text{Control}}_{\text{tumor weight}}\times 100\%.$$

### Immunohistochemistry

Xenograft tumors were ﬁxed in 4% paraformaldehyde (PFA), embedded in parafﬁn, and sectioned and stained with hematoxylin and eosin. Immunohistochemical staining of parafﬁn-embedded tumor tissues was performed using p62 (CST, 1:100 dilution), Ki-67 (CST, 1:100 dilution), Beclin-1 (CST, 1:100 dilution), Caspase-3 (CST, 1:100 dilution), Caspase-9 (CST, 1:100 dilution), Bcl-2 (CST, 1:100 dilution), Cyt-c (CST, 1:100 dilution), and LC3-II (CST, 1:100 dilution) primary antibodies and the ABC Elite immunoperoxidase kit in accordance with the manufacturers’ instructions. The method for immunohistochemical analysis of MVD was as follows: each section of each slice was randomly selected at least three fields of vision under 200× light microscope for picture taking. CD34 was expressed in vascular endothelial cells and was positive for brown granules in the cytoplasm of vascular endothelium. On the basis of Weidner’s and other proofing methods, the whole slice was scanned under 100× light microscope to obtain three high-density areas of blood vessels, namely, “hot spots” (HP). Then, the number of blood vessels with positive staining was counted under 200× light microscope. Microvascular identification did not require a complete luminal and erythrocyte when obvious vascular endothelial cells could be stained and separated from the blood vessels, tumor cells, and interstitial components. The number of vessels in the three fields was counted as the MVD value (number/HP).

All animal experiments complied with ethical regulations and were approved by the Laboratory Animal Center of Fujian Medical University (Tian et al., 2019).

## Results

### Quantified Components and Inhibitory Effect of SDEA on the Proliferation of Colorectal Cancer Cells

**Figure 1:**
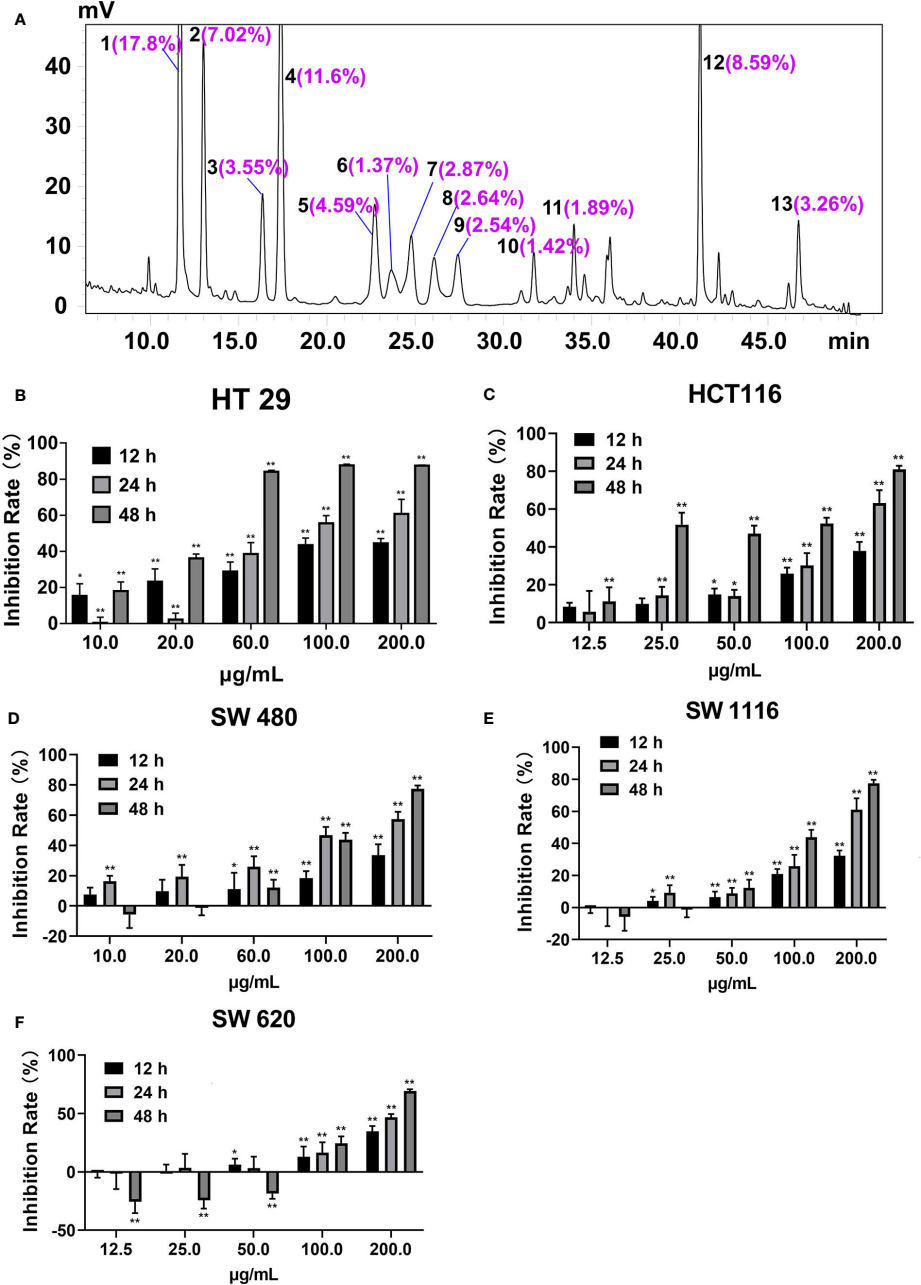
Peak area ratios of the main components of SDEA and its effect on colon cancer cell lines. **(A)** Peaks 1–13, amentoflavone; robustflavone; 2′′,3′′-Dihydro-3′, 3′′′-biapigenin; 3′,3′′′-binaringenin; delicaflavone; unknown; hinokiflavone; 2,3-dihydrohinokiflavone; chrysocauloflavone I; 2,3-dihydroisocryptomerin; robustaflavone 7,4′-dimethyl ether; heveaflavone; and 7,4′,7′′,4′′′-tetra-O-methyl-amentoflavone, respectively. Peak area ratios were calculated from HPLC chromatography of SDEA. **(B–F)** Inhibitory effect of SDEA on five colon cancer cell lines, The concentration of SDEA in HCT116, SW1116 and SW620 cells was 12.5–200 μg/ml, while that of HT29 and SW480 cells was 10–200 μg/ml, n ≥ 5, *p < 0.05; **p < 0.01, vs. control group, respectively.

There were 20 flavonoid components enriched in SDEA (Li et al., 2014; Yao et al., 2017), and 13 components were quantified, as shown in **Figure 1A**.

The inhibition rates of SDEA on the proliferation of HT29, HCT116, SW1116, SW480, and SW620 cells are shown in **Figures 1B–F**, with IC_50_ values of 17.43 ± 2.12, 20.13 ± 3.11, 78.12 ± 5.09, 63.09 ± 1.09, and 65.24 ± 2.65 μg/mL (mean ± SD, 48 h), respectively. The inhibition on the five colorectal cancer cell lines demonstrated a certain time and concentration dependence. The inhibition on HT29 and HCT116 cell lines were more remarkable than that on the other three cell lines, indicating that the two cell lines were more sensitive to SDEA.

### Morphological Changes Induced by SDEA

**Figure 2:**
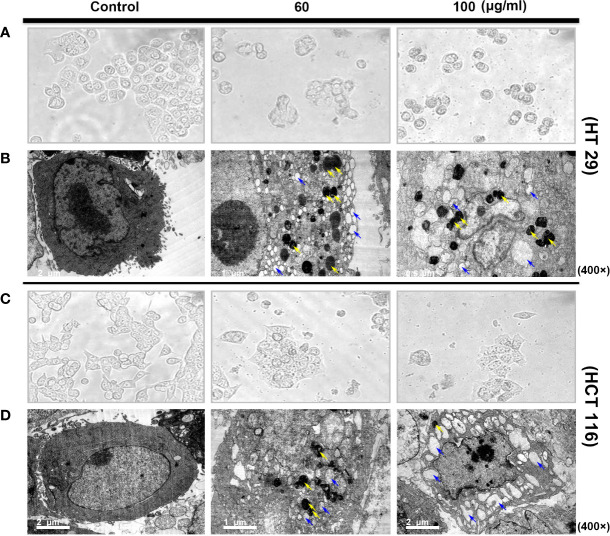
Morphological structure **(A, C)** and ultrastructural changes [**(B, D)** TEM images of autophagosome and autolysosome cells] in human colon cancer cells treated with SDEA under different concentrations for 24 h.

Observation *via* inverted microscope showed that the two colorectal cells had a normal polygonal shape and an adherent growth. After 24 h of intervention with different of SDEA concentrations (60 and 100 μg/mL), the cells gradually became round and floated up, and the intracellular granular material increased. The cells in the high-concentration group were lysed and then died, while the HCT116 cells were swollen (**Figures 2A, C**).

In addition, TEM observations showed more subtle differences between the two colorectal cells. The cells in the control group exhibited clear membrane integrity and surface-specific structure, complete organelles, uniform cytoplasm distribution, and normal nucleus and chromosomes. However, vacuolar-like structures and large numbers of vesicles encapsulated by two or more membranes with very high electron densities (dark staining, i.e., autophagic or autophagic lysosomes) were observed in HT29 cells after drug intervention. Significant vacuolar-like structures and several high electron density vesicles were also observed in HCT116 cells. The two colon cells showed a considerably increased number of vacuoles in the cytoplasm. Nuclear membrane disintegration occurred as the drug concentration was increased to 100 μg/mL, especially in HCT 116 cells (**Figures 2B, D**).

### Changes in MMP (△Ψm)

**Figure 3:**
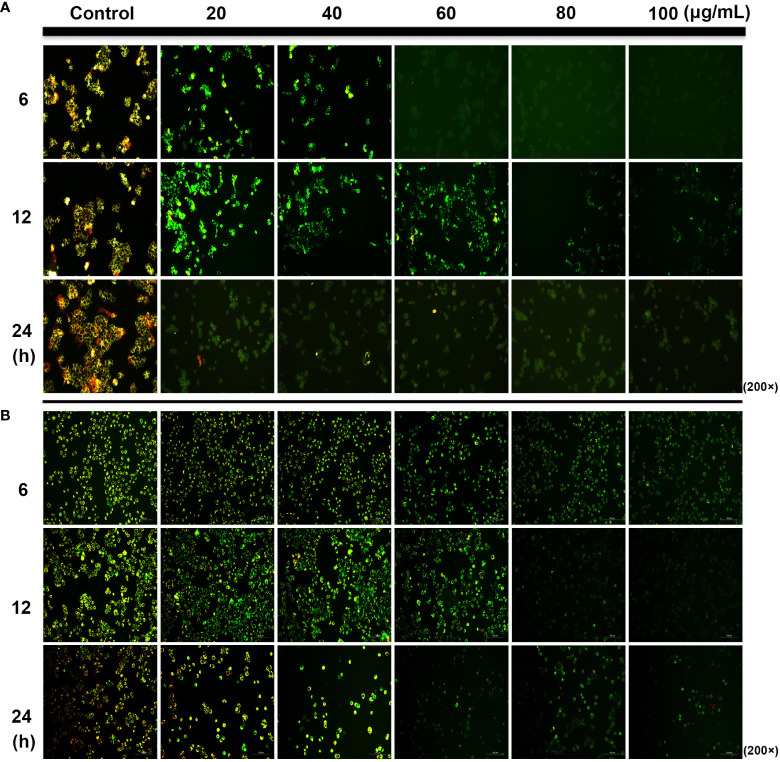
Change in the mitochondrial membrane potential of HCT116 **(A)** and HT29 **(B)** cells treated with different SDEA concentrations at different times.

Mitochondria plays an important role in the release of cytochrome C and apoptosis-inducing factor membrane proteins during apoptosis. Studies have shown that SDEA obviously causes the dissipation and collapse of MMP in HCT116 and HT29 cells in a concentration- and time-dependent manner. The phenomena shown in **Figures 3A, B** were as follows: the control cells at different time points (6, 12, and 24 h) emitted strong red and green fluorescence (merged as yellow fluorescence). With the increase in SDEA concentration and prolonged treatment time, the red fluorescence gradually weakened, whereas the green fluorescence remained bright or declined as the cell number decreased due to high SDEA concentration. When this concentration was increased to 80 μg/mL, the green fluorescence nearly disappeared.

### SDEA Induces Apoptosis of Human Colorectal Cancer Cells

During early apoptosis, phosphatidylserine (PS) is translocated from the cytosolic side of the plasma membrane to the cellular surface. This translocation exposes PS to the extracellular environment with the plasma membrane being left intact.

**Figure 4:**
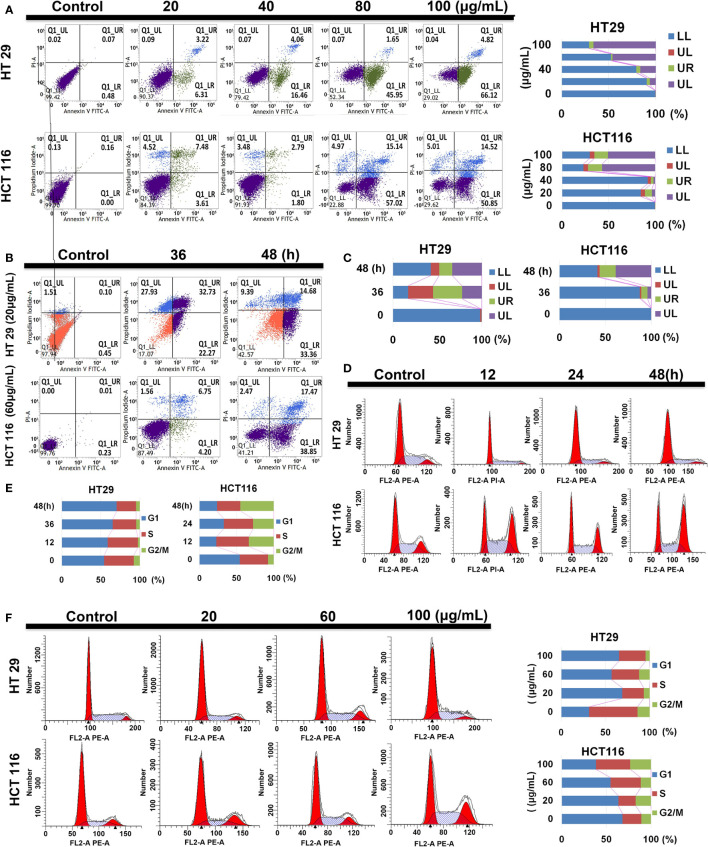
Effect of SDEA on apoptotic rate and cell cycle analysis. **(A)** apoptotic rate of different concentration-treated HT29 and HCT116 cells for 48 h. **(B, C)** apoptotic rates of HT29 and HCT116 cells treated with 20 and 60 μg/mL of SDEA at different times (*n* = 3), respectively. **(D, E)** Cell cycle analysis of HT29 and HCT116 cells treated with 100 μg/mL of SDEA at different times, respectively. **(F)** Cell cycle analysis of HT29 and HCT116 cells treated with different SDEA concentrations.

Compared with the control group, HT29 and HCT116 cells showed increased apoptotic ratio under SDEA intervention. With the increase in drug intervention concentration and the prolongation of intervention time, the proportion of early (UR Q3 region) and late (LR Q4 region) apoptotic cells increased in both cells, especially in HT29 cells. Under the same conditions, SDEA induced more apoptosis in HT29 cells than in HCT116 cells. In addition, HT29 cells were more sensitive to the changes in drug concentration and intervention time than HCT116 cells (**Figures 4A–C**).

### Effects of SDEA on Cell Cycle Arrest

The cell cycle distribution of the extract-treated HT29 and HCT116 cells was examined using ﬂow cytometry with propidium iodide (PI) DNA staining to further investigate the mechanisms responsible for the anti-proliferative effects of SDEA. The results showed that SDEA obviously increased the proportion of cells in the G1 phase and reduced that of cells in the G2/phase of HT29 cells when treated with 100 μg/mL under different concentrations and durations of intervention after 48 h. However, HCT116 cells performed very differently from HT29 cells (e.g., decrease in the G1 phase and significant increase in the S and G2/M phases; **Figures 4D–F**).

### SDEA Modulates Autophagy- and Apoptosis-related Proteins

**Figure 5:**
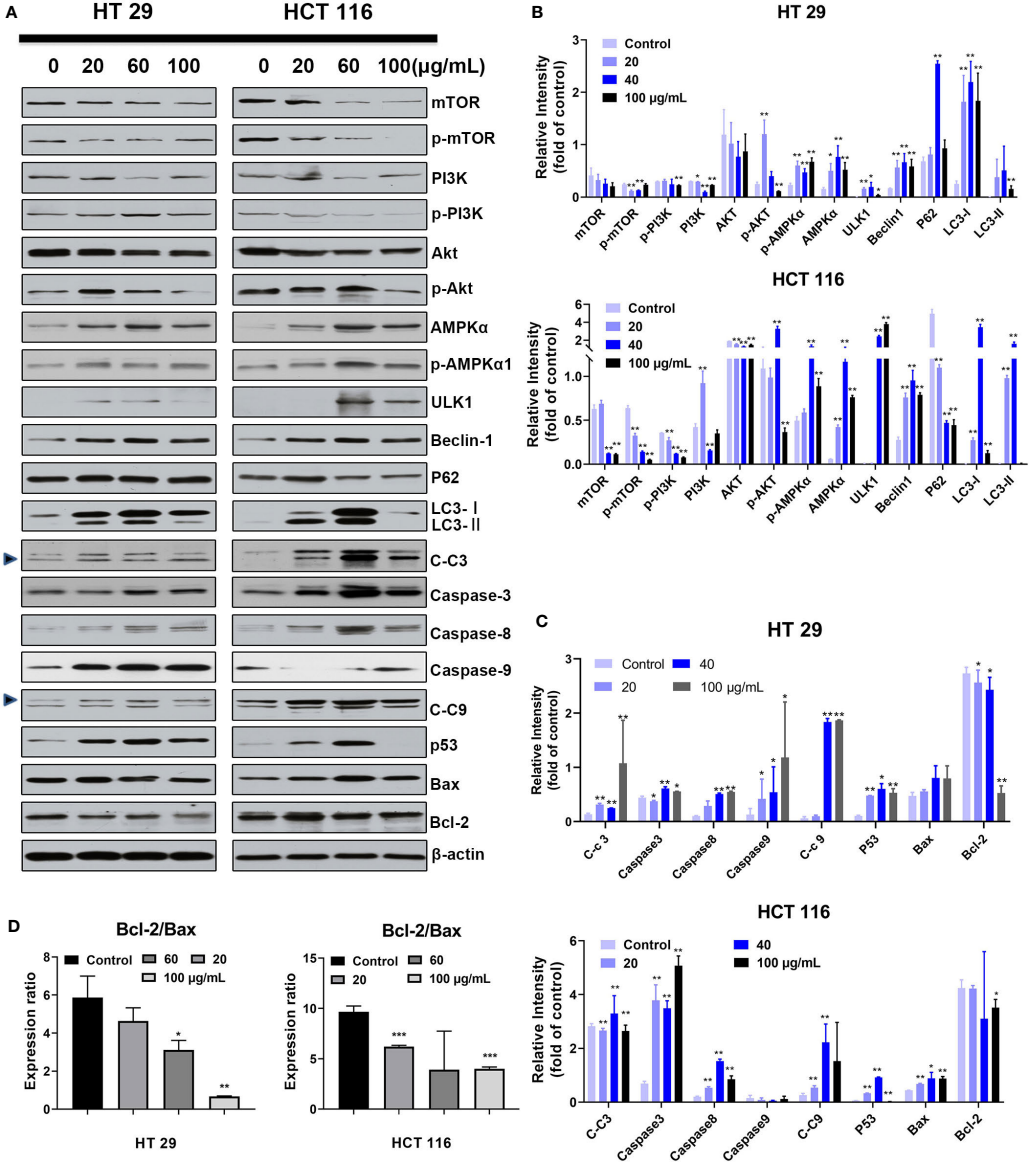
Effect of SDEA on the expression levels of apoptosis- or autophagy-related key nodes induced by it. **(A–C)** dose effect of SDEA on the expression levels of apoptosis and autophagy-related proteins in HT29 or HCT116 cells (*n* = 3, *p < 0.05; **p < 0.01, vs. control group. C-c3 means cleaved-caspase-3, C-c9 means cleaved-caspase-9). **(D)** Ratios of Bcl-2/Bax induced by different SDEA concentrations for 24 h.

Given the cell morphologic (increased autophagic bodies) and measurement data of translocated PS, the expression levels of key nodes related to apoptosis and autophagy pathways must be quantified and qualified. Thus, the cells were treated with different SDEA concentrations (0, 20, 60, and 100 μg/mL) for 24 h. After drug intervention, the expression levels of autophagic-related proteins mTOR, PI3K, and Akt decreased, while AMPKα, p AMPKα, ULK1, and Becline 1 increased in varying degrees as the concentration increased (**Figures 5A, B**). The autophagic key node P62 expressed in HT29 cells also increased in HT29 cells but decreased in HCT116 cells, indicating that sustainable autophagy oc curred in HT29 but was inhibited in HCT116. In addition, the expression levels of the key nodes in the apoptosis pathway, such as Caspase 3, 9, and 8, increased (**Figures 5A, C**), while the ratio of Bcl 2/Bax decreased (**Figure 5D**), indicating that apoptosis was initiated and the degree of apoptosis in HT29 was different from that in HCT116.

### Effects of SDEA on the Level of Autophagy-Related mRNA

**Figure 6:**
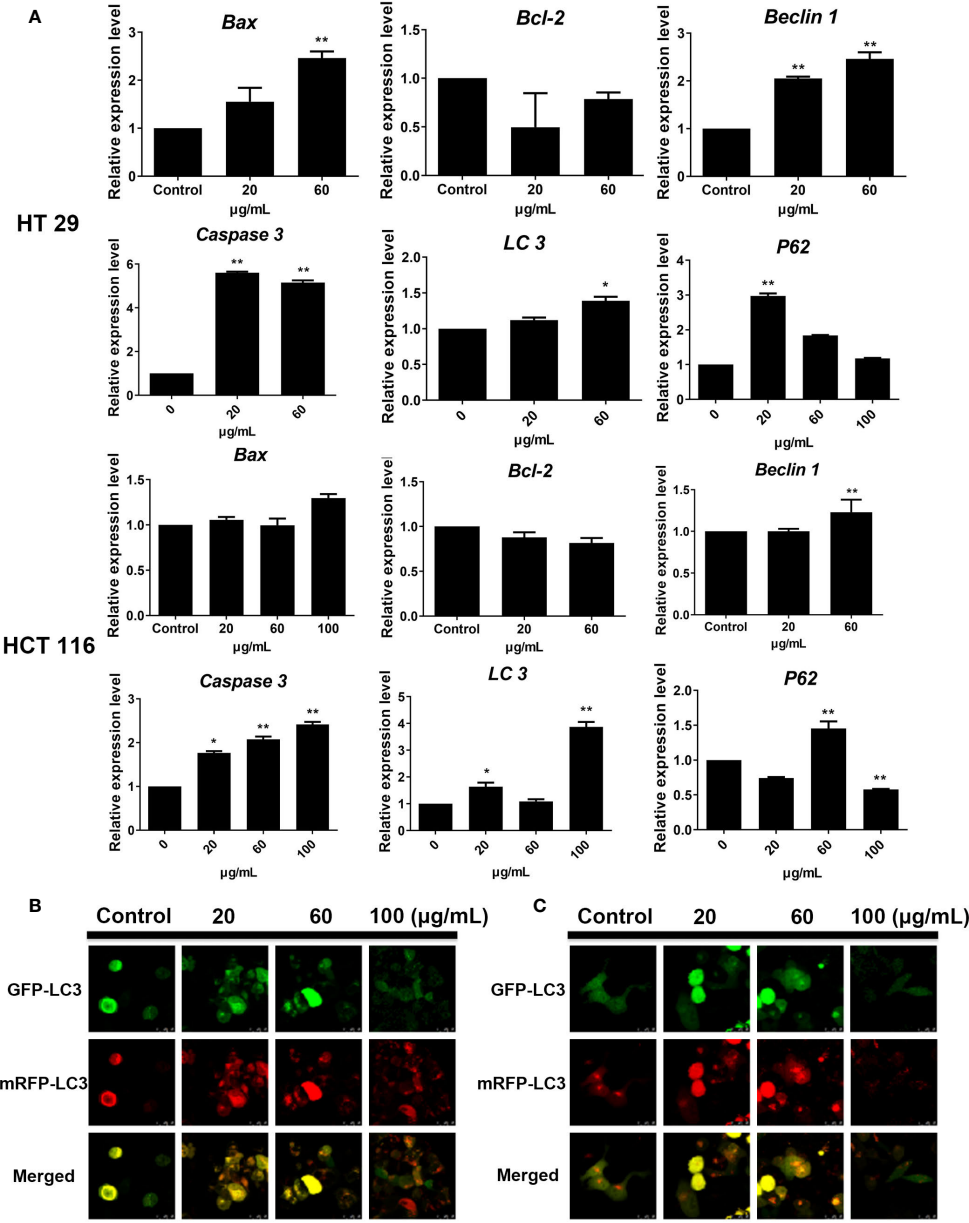
Effect of SDEA on the expression levels of apoptosis-related genes in cells and the GFP and mRFP signals of tandem ﬂuorescent LC3. **(A)** effect of SDEA on the mRNA of apoptosis-related genes *(Caspase-3*, *Bax*, and *Bcl-2*) and autophagy-related genes (*LC3*, *P62*, and *Beclin-1*) in HT29 and HCT116 cells, respectively, *p < 0.05; **p < 0.01, vs. control group. **(B, C)** GFP and mRFP signals of tandem ﬂuorescent LC3 (tfLC3, mRFP-GFP-LC3), respectively, showing autophagic fluxes in HT29 and HCT116 cells transfected with adenovirus expressing tfLC3 for 48 h and treated with different SDEA concentrations.

Gene amplification results showed that the expression levels of autophagy-related genes *LC3* and *Beclin-1* in HT29 and HCT116 cell lines were upregulated after SDEA intervention at different concentrations compared with those in the control group. In addition, the expression levels of apoptosis-related genes *Caspase 3* and *Bax* were gradually increased with the increase in SDEA concentration. The expression of *P62* in HT29 was significantly upregulated but decreased with the increase in SDEA concentration. However, this expression in HCT116 cell slightly increased and then significantly decreased as the level of intervention increased compared with that in the control group (**Figure 6A**). These results and the Western blot results, which showed the expression of apoptosis- and autophagy-related genes, were used to confirm the data above.

### Effects of SDEA on Autophagic Fluxes

The GFP signal is sensitive to the acidic and/or proteolytic conditions of the lysosome lumen, whereas mRFP is more stable. Therefore, colocalization of GFP and mRFP signals indicate a compartment, such as an phagophore or an autophagosome, that has not fused with a lysosome. By contrast, an mRFP signal without GFP corresponds to an amphisome or an autolysosome. After the HT29 and HCT116 cells were transfected with adenovirus carrying mRFP-GFP-LC3, the fluorescent protein in the control group was well expressed, and red and green fluorescence were evenly distributed throughout the cytoplasm with few punctuate dots. After the SDEA treatment, the “green only” dots and “red only” dots increased as the drug concentration was increased. The “yellow only” dots (where the yellow signal resulted from merging the red and the green fluorescence), the characterization of autophagosomes, also increased. Therefore, the extract inhibited HT29 and HCT116 cell proliferation by enhancing the autophagic flux in a dose-dependent manner (**Figures 6B, C**, respectively).

### Effects of SDEA on Tumor Growth and Xenograft Tumor Dedifferentiation

**Figure 7:**
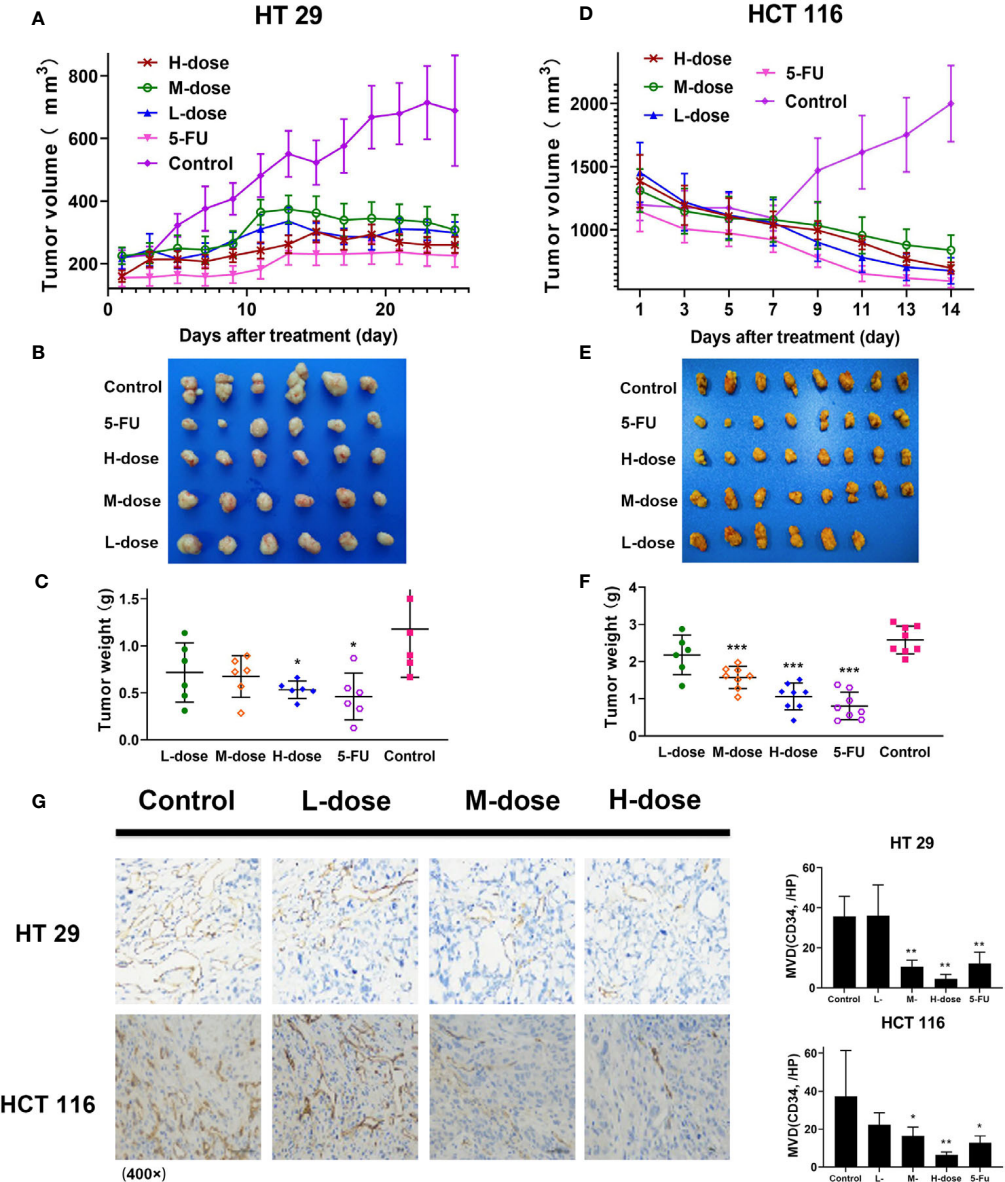
Antitumor effect of SDEA *in vivo*, immunohistochemistry analysis, and MVD counts of xenograft tumor tissues in colon tumors. **(A, D)** Tumor volume in HT29 (*n* = 6) and HCT116 (*n* = 8), respectively, mean ± SEM; **(B, E)** images of tumor lump removed during the last treatment day; **(C, F)** weight of tumor lump removed during the last treatment day **(B, C)** (HT29), **(E, F)** (HCT116), mean ± SEM, HT 29 (*n* = 6) and HCT 116 (n = 8), respectively (*p < 0.05; ***p < 0.001, vs. control group). **(G)** Effect of SDEA on the expression of CD34 staining (× 400); H, MVD counts (*n* = 6, *p < 0.05; ** *p* < 0.01; 5-FU, i.v., 5 mg/kg/2 days; control, po., N.S.; low dose, po., 100 mg/kg/day; medium dose, po., 200 mg/kg/day; and high dose, po., 300 mg/kg/day).

In this part, SDEA showed an effective anti-colon cancer effect in both colorectal xenograft tumors *in vivo*. Its inhibition rates of HT29 tumor growth were 39.2% ± 9.2% (low-dose group), 42.8% ± 8.3% medium-dose group), 54.7% ± 3.5% (high-dose group), and 60.9% ± 9.2% (5-FU group, mean ± SEM, *p* < 0.05 vs. control group) without loss of mice body weight during 25 days of treatment. The inhibition rates of HCT116 tumor growth were 15.6% ± 8.8% (low-dose group), 39.0% ± 3.7% (medium-dose group), 58.9% ± 4.9% (high-dose group), and 68.8% ± 5.4% (5-FU group, mean ± SEM, *p* < 0.001 vs. control group) *in vivo* without loss of mice body weight for 14 days (**Figures 7A–F**).

Immunohistochemical microvascular density (MVD) analysis was used to analyze the transplanted tumors. The results showed that in HT29 and HCT116 cell-transplanted tumors, the positive rate of CD34 in the control group was significantly higher than that in the drug treatment group, and the positive rate in the high-dose group (300 μg/kg) was the lowest, decreasing in a dose-dependent manner (**Figure 7G**). The results also showed that SDEA decreased the MVD counts of HT29 xenografts as follows: 34.0 ± 1.7/HP (low-dose group), 41.6 ± 3.2/HP (model group), 29.1 ± 2.6/HP (medium-dose group, *p* < 0.05 vs. model group), and 20.0 ± 4.2/HP (high-dose group, *p* < 0.01 vs. model group). For HCT116, the MVD counts decreased among the groups as follows: 6.4 ± 1.5/HP (low-dose group, *p* < 0.01 vs. model group), 15.1 ± 3.4/HP (medium-dose group, *p* < 0.05 vs. model group) and 22.3 ± 6.3/HP (high-dose group, *p* < 0.05 vs. model group). Therefore, SDEA obviously reduced the MVD of xenografts in a dose-dependent manner.

**Figure 8:**
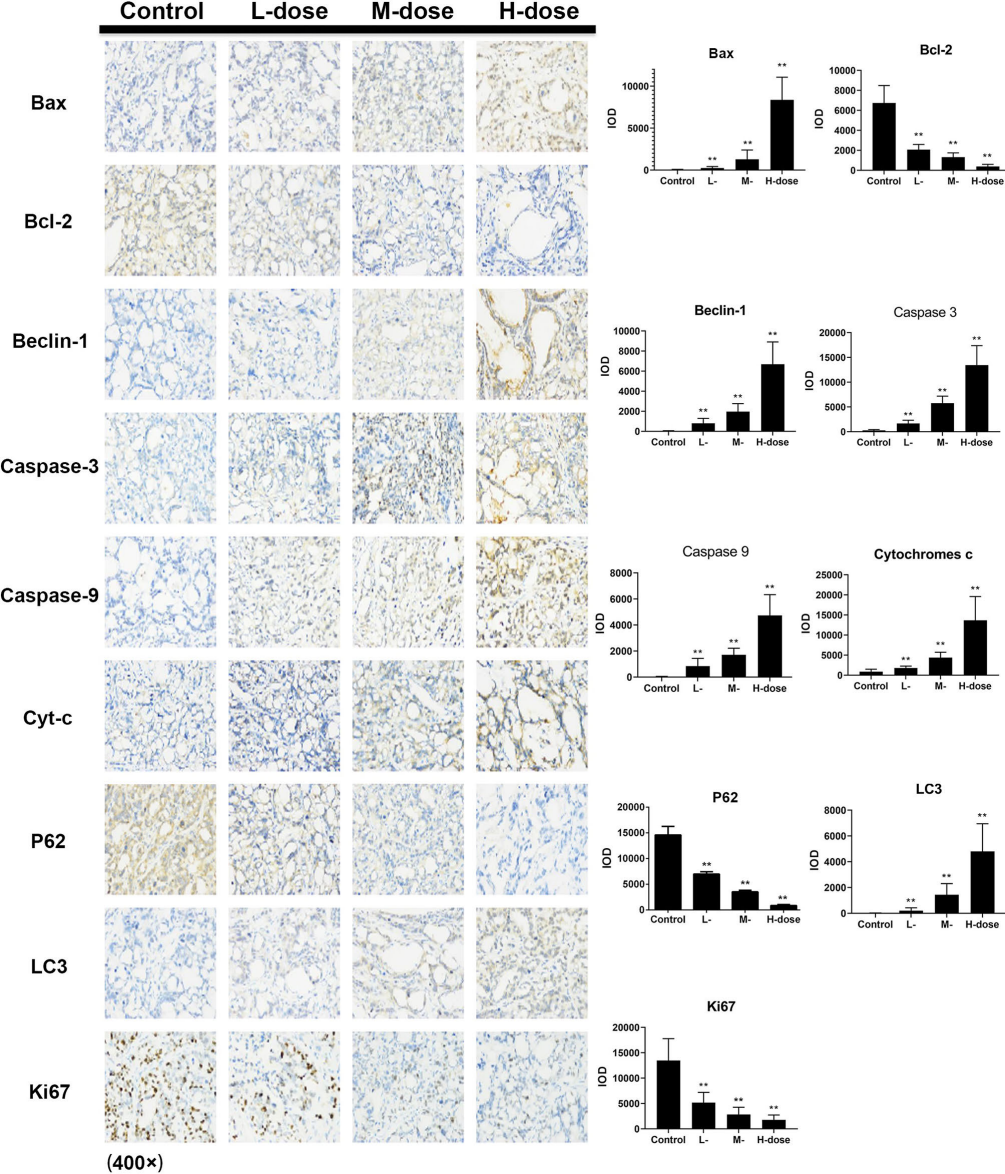
Effect of SDEA on the expression levels of apoptosis- and autophagy-related genes in the issue from HT29 xenografts (control, po., N.S.; low dose, po., 00 mk/kg/day; medium dose, po., 200 mg/kg/day; and high dose, po., 300 mg/kg/day). (*p < 0.05; **p < 0.01, vs. control group).

**Figure 9:**
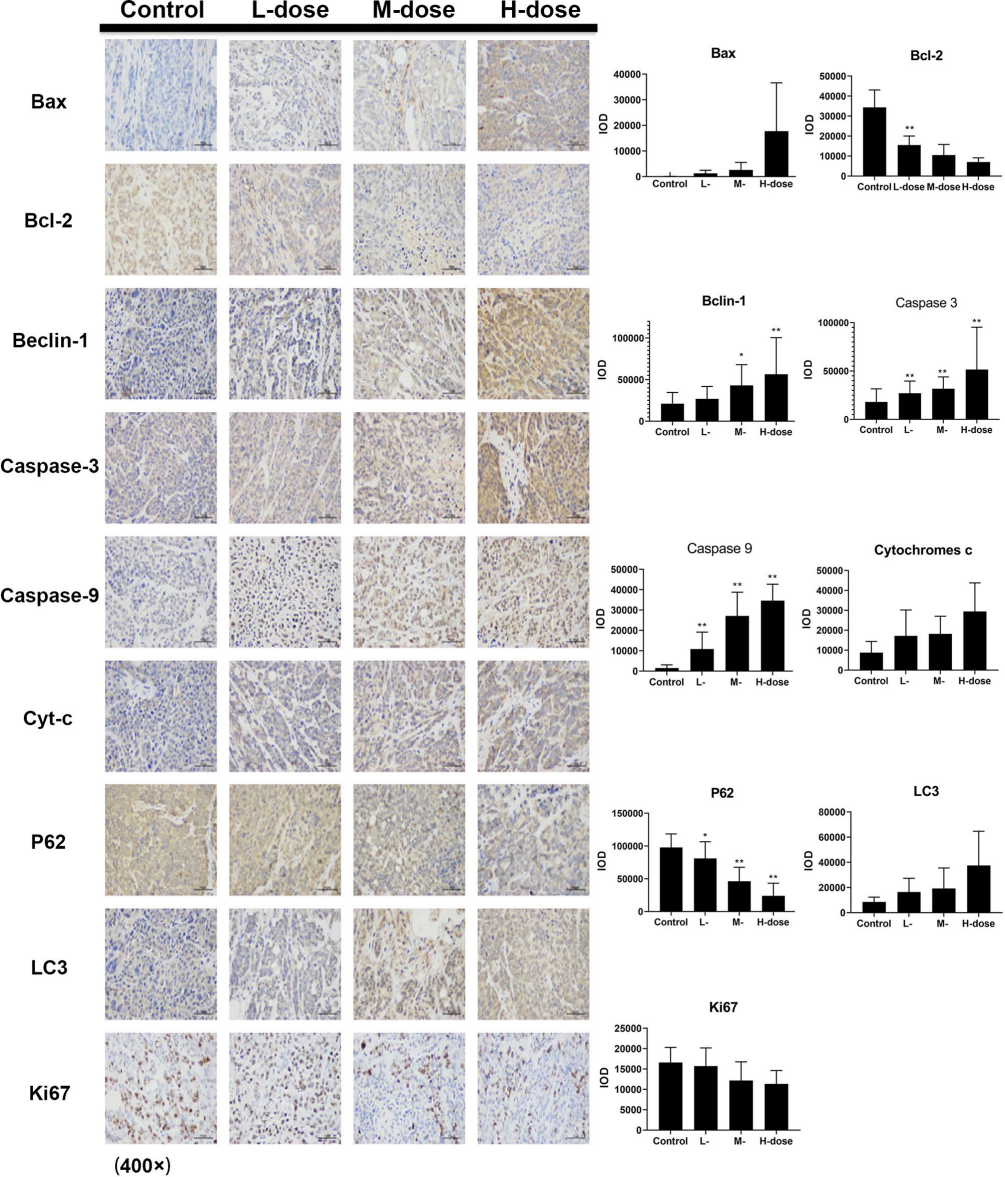
Effect of SDEA on the expression levels of apoptosis- and autophagy-related genes in the issue from HCT116 xenografts (control, po., N.S.; low dose, po., 100 mg/kg/day; medium dose, po., 200 mg/kg/day; and high dose, po., 300 mg/kg/day). (*p < 0.05; **p < 0.01, vs. control group).

The immunohistochemical results for HT29 and HCT116 cells (**Figures 8** and **9**, respectively) showed that in both transplanted tumors, the expression levels of cell apoptosis- and autophagy-related key nodes and cell proliferation antigen *Ki67* significantly changed in the treatment groups, especially in HT 29 cells, compared with the control group. Among these key nodes, the expression levels of pro-apoptotic related genes *Cyt-c*, *Bax*, and apoptotic executive genes *Caspase-3*, *9* increased with the increase in treatment dose. Compared with the control group, the expression of apoptotic suppressor genes decreased with the increase in treatment dose, especially in the high-dose group (*p* < 0.01). A significant difference in apoptotic suppressor genes was found between the low-dose group and the high-dose group (*p* < 0.05) and between the middle-dose group and the high-dose group (*p* < 0.01). The expression of Ki67 significantly decreased with the increase in treatment dose (*p* < 0.01) compared with the control group. The expression levels of LC3 and Beclin-1 increased with the increase in treatment dose (*p* < 0.05) compared with the control group, whereas the expression of autophagy inhibitor P62 decreased (*p* < 0.05) in the high-dose group (*p* < 0.01).

## Discussion

The results showed that SDEA had a significant growth inhibitory effect on colon cancer cells, especially on HT29 and HCT116 cells. As shown by the changes in cell morphology and structure after drug intervention, the number and volume of vacuole-like structures in HT29 cells gradually increased with the increase in drug concentration and prolongation of action time. Subcellular morphological analysis by transmission electron microscopy showed that increasing SDEA induced a large number of apoptotic bodies and autophagosomes in HT29 and HCT116 cells and most organelles were lost in these cells. These results indicated that inducing apoptosis and autophagy in colon cancer cells could be the two possible pathways of the mechanisms of SDEA inhibiting cell proliferation. As a form of programmed cell death, apoptosis has typical morphological characteristics and clear biochemical mechanism compared with other types of cell death. In this study, Annexin V-FITC/PI was used to stain the cells, and flow cytometry was used to detect apoptosis. The results showed that SDEA could significantly induce the apoptosis of HT29 and HCT116 cells in a concentration- and time-dependent manner.

As a fluorescent probe used to detect MMP, JC-1 (5, 5′, 6, 6′-tetrachloro-1′, 1′, 3, 3′-tetraethyl-imidacarbocyanine iodide) showed that SDEA could significantly reduce the MMP in HT29 and HCT116 cells and even disintegrate it. Mitochondrial permeability transition (MPT) acts as an endogenous activation pathway that induces apoptosis. The change in MPT is an indicator of mitochondria during apoptosis; it alters the balance between intracellular and extracellular ions, leading to decreased or even disintegrated △Ψ_m_. Relevant studies have shown that almost all apoptotic cells are accompanied by changes in △Ψ_m_. In the present study, the changes in △Ψ_m_ in HT29 and HCT116 cells could be detected in a short time using high SDEA concentration, but the fluorescence signal was relatively weak. The higher the concentration, the weaker the fluorescence signal was. On the one hand, the growth status of cells deteriorated rapidly because of high drug concentration, which directly affected the entry of JC-1 into the cells. On the other hand, the increase in drug concentration interfered with the fluorescent probe and changed its aggregation state, thus affecting the intensity of the fluorescent signal.

The changes in cell biochemistry, morphology, and structure of cell cycle and the transformation of adjacent periods are all under the strict control of the cell itself and environmental factors. Conversions from G1 to S and from G2 to M are the two key nodes in cell cycle regulation. Different proteins and polypeptide factors could regulate cell cycle at a multi-factor level after acting on these regulatory points. In this study, cell cycle arrest in HT29 and HCT116 cells by SDEA was detected. The results also showed that a significant difference in HCT116 cell cycle arrested in the G2/M phase was found after 48 h of drug intervention and blocking of HT29 cell cycle transition from G1 phase to S phase compared with the control group. In HT29 cells, a large proportion of cells were in the G1 phase, while the proportion of S phase cells decreased gradually with the increase in drug concentration. The different stages of cell cycle corresponded to different regulatory factors or cyclins. Cyclin A, C, D, and E were expressed in the G1 phase. The latter three proteins were limited to the G1 phase. They began to degrade when they entered the S phase and only played a role in the process of conversion from G1 phase to S phase. Cyclin D is necessary for cell transformation in the G1/S phase. In addition, the HCT116 cells arrested in the G2/M phase increased, whereas both cells in the G1 phase significantly decreased. This result demonstrated that the cells that replicated DNA were blocked before the subsequent mitotic phase and mitosis active proteins, such as Wee1, CylinB1, and CDK1, may be influenced by the gradual increase in SDEA concentration.

This study exhibited that SDEA could block the cell cycle of HT29 cells in the G1 phase, which may be related to the regulation of cyclins, and that of HCT116 cells in the G2/M phase, which indicated that the mitosis active proteins were suppressed by SDEA.

Meanwhile, SDEA could enhance the expression levels of apoptosis-related genes *Caspase-3, Caspase-8, Caspase-9, p53*, and *Bax* and inhibit the expression of apoptotic inhibitor gene *Bcl-2*. The proportion of *Bcl-2/Bax* significantly decreased with the increase in SDEA concentration. The expression of *Caspase-3* in the treatment groups was significantly upregulated compared with that in the control group. In addition, the expression levels of *Cleaved-Caspase-3* and *Cleaved-Caspase-9* splices significantly increased in the treatment groups, indicating that *Caspase-3* and *-9* were activated to promote or execute cell apoptosis. SDEA induced the expression levels of apoptosis-related genes in HT29 and HCT116 cells in a concentration-dependent manner. As mentioned above, cell stress or apoptotic signal could induce the mitochondria to release *Cyt-c*. As an apoptotic-inducing factor, *Cyt-c* can form complexes with apoptosis-related regulatory genes, activate apoptotic executive genes, trigger caspase gene cascade reaction, and induce apoptosis. Therefore, SDEA could induce the expression levels of apoptosis-related genes and the decline of MMP, which is a continuous process, ultimately leading to apoptosis in HT29 and HCT116 cells.

In **Figure 5A**, p-mTOR/mTOR, p-PI3K/PI3K, p-AKT/AKT, and p-AMPKα/AMPKα expressions in two different cancer cells are in accordance with certain rules, but not completely in accordance with the concentration dependence, especially in low or middle dose groups. As we know, apoptosis and autophagy are in competition to dominate the process of cell death. At low concentration, apoptosis is dominant in cell death. With the increase of concentration, autophagy gradually takes the dominant position, whereas intense autophagy leads to cell death induced by high concentration.

*ULK1, P62, Beclin-1*, *LC3-I*, and *LC3-II* are key regulatory genes in the process of autophagy. Detecting the expression levels of ULK1, P62, Beclin-1, and LC3-II could determine the level of autophagy. SDEA could also induce the expression levels of these regulatory key proteins and genes; upregulate the expression levels of ULK1, Beclin-1, LC3-I, and LC3-II; and inhibit the expression of P62 in HT29 and decrease it in HTC116 in a concentration-dependent manner. Thus, SDEA could induce autophagy in HT29 and HCT116 cells by inhibiting the AMPKα-dependent pathway.

The intensity of autophagic flow is an important index that measures the level and patency of autophagy. During the autophagy process, LC3 molecule is located on the autophagic membrane; it encapsulates the substrate enters the lysosome, which eventually degrades it. After *LC3* was labeled with RFP and GFP, the occurrence of intracellular autophagy could be clearly observed. The results showed a certain level of autophagy, which contributed to cell growth, in the control group. After SDEA intervention, the level of autophagy increased gradually with the increase in drug concentration. Yellow fluorescence (red and green fluorescence superpositions) spots and red fluorescence spots increased. The red fluorescence spots significantly increased in the intervention group with increased drug concentration, indicating that the level of autophagy also increased. Autophagic flux assay confirmed that SDEA could significantly enhance autophagy in HT29 and HCT116 cells. In addition, transmission electron microscopy showed that autophagic or autophagic lysosome was formed in the drug treatment group compared with the control group. Many vacuole structures and necrosis-like morphological changes were observed in the two cells in the higher concentration groups, further revealing that SDEA could induce apoptosis during autophagy of colon cancer cells. In conclusion, SDEA could significantly induce autophagy in HT29 and HCT116 cells, accompanied by apoptosis in a concentration-dependent manner.

In this study, nude mice xenograft tumor models were used to establish xenograft models of HT29 and HCT116 cell lines and explore the therapeutic mechanism of SDEA in colon cancer. No significant change was observed in the weight of mice during the treatment period, even in the high-dose group (300 mg/kg). This high dose could significantly inhibit the growth of xenograft colorectal tumor (the inhibition rates for HT29 and HCT116 cells were 54.7% ± 3.5% and 58.8% ± 4.9%, respectively), both lower than 5-FU (60.9% ± 9.2% and 68.8% ± 5.4%, respectively).

Immunohistochemistry was used to detect the expression levels of apoptosis- and autophagy-related proteins Cyt-c, Caspase-3, Caspase-9, Bax, Bcl-2, Beclin-1, P62, and LC3 in the two xenograft models. The results showed that SDEA could significantly induce changes in the expression levels of the key regulatory genes, leading to apoptosis and autophagy in tumors and the inhibition of tumor growth. CD34 antigen was used as a marker molecule of the blood vessel in tumor tissues to count blood vessel density. The expression of CD34 in the drug treatment groups was significantly lower than that in the control group, and the microvessel density in the corresponding tumor tissues was smaller. This finding indicated that SDEA could inhibit angiogenesis in the two cell-transplanted tumor tissues, thus affecting tumor growth. Furthermore, the percentage of Ki67-positive cells was used to judge the proliferation degree or cell proliferation activity of the tumor cells. The results demonstrated that SDEA could significantly inhibit the expression of Ki67 in tumor tissues. The inhibitory effect of SDEA was dose-dependent compared with that in the control group. In conclusion, SDEA inhibited the growth of HT29 and HCT116 xenograft tumors in nude mice by inducing the expression levels of apoptosis- and autophagy-related genes, inhibiting the formation of neovascularization in transplanted tumors, and reducing the proliferation activity of these tumors.

## Conclusion

SDEA has significant anti-colon cancer effects on HT29 and HCT116 cells *in vitro* and *in vivo*, indicating that this extract possessed a good potential as an anti-colon agent. The anti-colon cancer mechanisms of SDEA could be deduced as simultaneously inducing cell autophagy and apoptosis in colorectal carcinoma *via* the AMPKα- and caspase-dependent signaling pathways.

## Data Availability Statement

All datasets generated for this study are included in the article.

## Ethics Statement

The animal study was reviewed and approved by Fujian Medical University Laboratory Animal Centre.

## Author Contributions

HY and XL conceived and designed the experiments. SGL, XW, and PS contributed to analyzed the data and prepared all the figures and wrote the manuscript. GW, SLL, DX, and BC contributed to the experimental procedures. AL and LH provided technical support. All authors contributed to the article and approved the submitted version.

## Conflict of Interest

The authors declare that the research was conducted in the absence of any commercial or financial relationships that could be construed as a potential conflict of interest.
