# Supplementary figures and images for: Ethyl Acetate Extract of Selaginella doederleinii Hieron Induces Cell Autophagic Death and Apoptosis in Colorectal Cancer via PI3K-Akt-mTOR and AMPKα-Signaling Pathways
Source: Front Pharmacol. 2020 Oct 19;11:565090. doi: 10.3389/fphar.2020.565090 (PMC7604386; doi:10.3389/fphar.2020.565090)

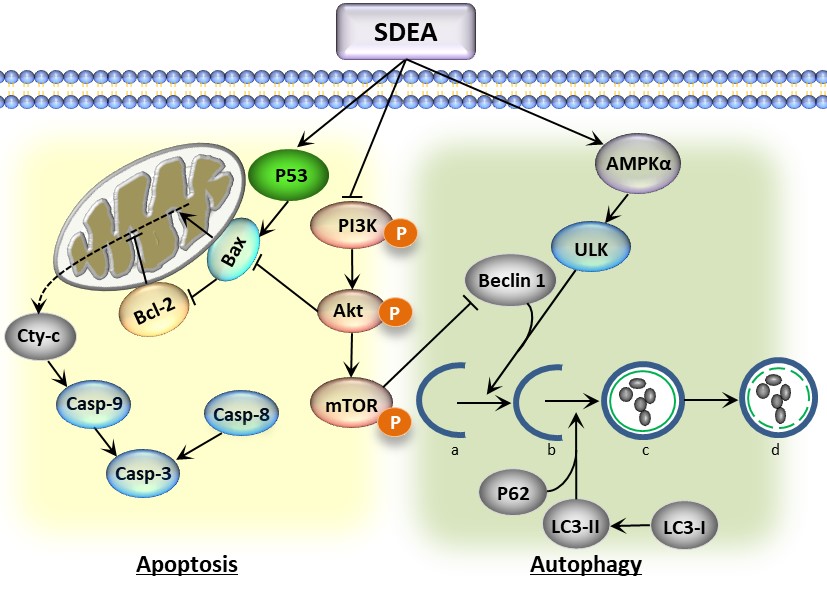

Supplement: Supplementary file 1 [file Image_1.jpeg]
